# The Duties and Responsibilities of the Physician

**Published:** 1864-12

**Authors:** J. B. Hoag


### THE DUTIES AND RESPONSIBILITIES OF THE PHYSICIAN.

AN ESSAY READ BEFORE THE STARK CO. MED. SOC., OCT. 1, 1864.

By J. B. HOAG, M. D.

*Mr. President and Gentlemen:*—

The task which has been assigned me is one that well might employ an abler pen, and which one, far more competent, might well enter upon with hesitancy and reluctance. The first item which I shall notice in connection with the first department of my subject is, *The duty of being amply qualified for the vocation of his choice.*

Before entering fully upon the discussion of the thought under consideration, I will premise that the young man who contemplates becoming a disciple of Esculapius, contemplates assuming a position, at once honorable, which has claimed for its votaries the noblest intellects, the most gigantic minds, of which the world can boast; but while this is true, it is a vocation fraught with grave responsibilities, not unfrequently vexatious perplexities, and onerous and fatigueing duties. Those who imagine it to secure a life of ease or indolence, will ascertain upon experiment, that they have counted without their host. There is, to me, something grand and sublime in being instrumental in mitigating the sufferings of those who are enduring the tortures of disease; in restoring to the society of those who are dear to them, by the ties of consanguinity or affection, those whom, to all human appearance, death has claimed as his victims; in restoring the roseate hue of health to the wan and cadaverous cheek of the invalid.

Having, after due consideration, and a full appreciation of the dignity and responsibility of the calling, as well as difficulties incident to it, decided upon making it a profession, the next idea is, the qualifications necessary to enable one to discharge the duties that will necessarily devolve upon him.

Life and health are the most desirable of earthly blessings, and when disease deprives us of the one, and threatens to wrest from us the other, we have the greatest interest possible at stake to induce us to seek aid from those who are justly competent to afford relief and guarantee to us those estimable boons, and the man, young or old, who presents himself before the public as one who is capable of administering to the necessities of the sick, without a consciousness of a competent qualification, is assuming a fearful responsibility.

The question, how can the requisite qualifications be obtained? is one that properly presents itself for consideration. We answer, by perusing the works of those who have preceded us, and who have left their experience upon record, for the benefit of their successors. The student of medicine should be diligent in seeking to acquire knowledge of this kind. He who purposes to assume the responsible position of administering to the diseased, and warding off, for the time being, the shafts of death from its intended victim, has no time for idleness but he should avail himself of every opportunity to acquire information, which is calculated to enable him to be of benefit to those to whom he may, in the future, be called upon to administer. Here it would be proper to remark, that there is a great difference between reading and studying. The text-books which it is necessary for the student of medicine to peruse, can not, with benefit, be read in a careless manner, as might be done with a novel or history, with the thoughts wandering to other subjects, but it must be *carefully*, *diligently studied*, and the ideas therein contained *permanently* stored in the mind and frequently made the subject of thought and re flection, in order that he may be able to reduce them to purposes of pıactical utility, whenever circumstances render it requisite for him so to do.

If there is, in times of peace, one curse that civilized society suffers more from than any other, it is quackery—empiricism. The vast number of valuable lives that have been sacrificed at the shrine of ignorant presumption, the number that have been materially injured for life, by ignorant pretenders to the art of medicine, can never be correctly estimated while time rolls its onward course to the vast ocean of eternity. The moral turpitude that is attached to this reprehensible course, is extreme. Compared with the empiric, the swindler, nay, highway robber, who takes but pelf, without injury to life or health, are harmless. These considerations ought to stimulate the student to every possible exertion to become, as far as possible, competent for his vocation. But he cannot learn all that is necessary to be known from books alone. He should have recourse to the lecture room, where, under the instruction of those who have previously climbed the weary steps of scientific knowledge, he may gain information, by ocular demonstration, that he can obtain from no other source. There are advantages to be derived from this method of instruction, which no other advantages possess. Ordinarily, deeper and more lasting impressions are received from oral instruction than is the case by perusing that which is written; and the lecture room affords opportunities for ocular demonstration in chemical experiments, natural and pathological anatomy, and clinical medicine which cannot be obtained from books alone. It is conceded by all that knowledge conveyed to the mind through the medium of the senses is from the nature of the mind more permanent than that obtained through any other medium.

The time has been when legislative enactments threw the shield of their protection round the public, and made it obligatory upon all would sustain the relation of physician to the public to emenate from colleges founded for their benefit, with vouchers of their ability; but this barrier against imposition has been broken down to too great an extent for the public good, but yet the moral obligations to be competently qualified, as well as the rights of our patrons, remains the same.

But the study and the lecture room do not afford all the facilities for obtaining information of value to the medical student. It has been well said, by an eminent medical writer, that "the theory of medicine can be learned from books and in the lecture room, but the practical knowledge necessary for success must be obtained by the bed-side of the sick, in the chamber of the invalid." It behooves the student, then, to avail himself of every opportunity that is offered to visit the sick, observe the symptoms of disease, and the action of remedies used.

After having become qualified to stand the scrutiny of a thorough examination, and by application over the "midnight oil," and years of studious application to medical books and all other available means calculated to afford the requsite information, to enter upon his vocation, the physician presents himself before the public as a candidate for public patronage, he ought not to regard himself as having arrived at the finality of his attainments, but rather as having merely laid the foundation on which it is his business for a life time to build. All through the course of his life he should be a student—a hard, devoted student—determined to dig deep into the hidden mysteries of the healing art, and avail himself of all the new discoveries which mark, with rapid strides, the progressive character of the age in which we live; that he may use them for the benefit of those to whose necessities he is called to administer.

But other duties devolve upon him. While it is his imperative duty to guard against needless exposure, which is calculated to jeopardize his health, no considerations of selfish indolence should ever prompt him to refuse to brave storms or tempests, or to endure fatigue, in the performance of professional duties. The physician ought ever to be willing to sacrifice his ease and comfort, if the necessities of the sick and suffering require him to do so. While we frankly admit it to be the duty of the physician to attend, when his services are required, upon the indigent and those who are proper objects of charity, irrespective of the certainty of an adequate compensation, we claim that he is not, in duty, bound to dispense his favors to those who *might*, but *will not*, make an effort in that direction.

The physician should regard himself as the guardian of the public health. It is his duty under all circumstances, and especially during the prevalence of epidemics, to give to his patrons such information as may serve for prophylactic purposes in the avoidance of disease.

The items which we have enumerated have reference particularly to the duties which the physician owes to his patrons, but there are others which refer particularly to the profession. In regard to these it may truthfully be said, that the golden rule which the Saviour enunciated for the guidance of His followers, is sufficient for a guide if strictly adhered to, in the conduct of one physician towards another. Courtesy, affability, a recognition of the rights of our professional brethren, should under all circumstances mark the course of every physician.

Much more might be said relative to the duties which devolve upon the physician, but suffice it to say, in conclusion, on this part of our subject, that they are varied and controlled to a great degree by circumstances, which require the exercise of a sound, deliberate judgment.

We now preceed to remark briefly in reference to the responsibilities which he incurs, and which are the legitimate results of his vocation.

A fearful responsibility rests upon the physician if bad results follow the treatment which the sick receives at his hands, provided, in consequence of ignorance or indolence, that treatment was not adapted to the case. I frankly confess that I can not imagine how those who profess to be skilled in the healing art, but know full well that they do not possess the requisite knowledge, can rest easy under the load their consciences must bear, with the consciousness of the amount of responsibility they are assuming. Quackery has doubtless been instrumental, to a great extent, in peopling the regions of perdition with many who, had not their lives been cut short by a false pretender, might have complied with the requisitions of the Gospel and have become heirs of salvation.

The physician assumes grave responsibilities in all the duties of his calling, but the extent of it is determined, in a great measure, by circumstances. He ought always to reel that while he maintains that relation to the public, a great amount of responsibility rests upon him, of which he can not divest himself. To him the anxious mother, the solicitous wife, the grieved father, the afflicted husband, and the weeping child, look with anxious solicitude, when their loved ones are prostrated with disease, and the grim monster threatens to snatch them from their loved embrace.

Other ideas might properly be noticed but our article has reached a greater length than we at first anticipated, and we will leave the subject for a future time, or more able hands.

